# The buccal fat pad graft in the closure of oroantral communications

**DOI:** 10.1016/S1808-8694(15)31397-5

**Published:** 2015-10-17

**Authors:** Marvis Allais, Paul Edward Maurette, André Luis Vieira Cortez, Jose Rodrigues Laureano Filho, Renato Mazzonetto

**Affiliations:** 1 Master’s degree in buccomaxillofacial surgery and trauma, FOP-UNICAMP. Doctoral student in buccomaxillofacial surgery and trauma. FOP-UPE; 2 Master’s degree in buccomaxillofacial surgery and trauma. FOP-UNICAMP, Doctoral student in buccomaxillofacial surgery and trauma. FOP-UPE; 3 Doctoral degree in buccomaxillofacial surgery and trauma. FOP-UNICAMP; 4 Doctoral degree in buccomaxillofacial surgery and trauma. FOP-UNICAMP. Associate professor of buccomaxillofacial surgery and trauma, Universidade de Pernambuco. Faculdade de Odontologia de Pernambuco. FOP-UPE. Recife - PE - Brasil; 5 Doutoral degree in buccomaxillofacial surgery and trauma, UNESP. Full professor of buccomaxillofacial surgery and trauma, Universidade de Piracicaba. Faculdade de Odontologia de Piracicaba. FOP-UNICAMP. Universidade de Pernambuco. Faculdade de Odontologia de Pernambuco

**Keywords:** fat body, oral fistula

## INTRODUCTION

Buccosinusal fistulae result from disease, trauma or minor surgery;^1^ the most common cause is extraction of upper molars, as their roots are close to the maxillary sinus.1 Surgery is needed for the closure of buccosinusal fistulae when they measure over 3 mm or if there is inflammation/infection of the maxillary sinus or periodontal area.^2^

Use of the buccal adipose body as a pedicle graft has become more frequent in buccomaxillofacial surgery, given its speed, relatively ease and high success rate. The first report of buccal reconstruction was made in 1977; however, Tidemann et al.^3^ only published a paper detailing the anatomy of the buccal adipose body, its blood supply, the surgical technique and the results of 12 cases of mouth defect reconstruction in 1986.

## CASE REPORT

A male patient aged 51 years was referred for the treatment of a buccosinusal fistula of six months duration after removal of the upper left second molar. The patient complained of pain, a bad taste in the mouth and a feeling of fluid in the nose after drinking any beverage.

**Figure 1:**
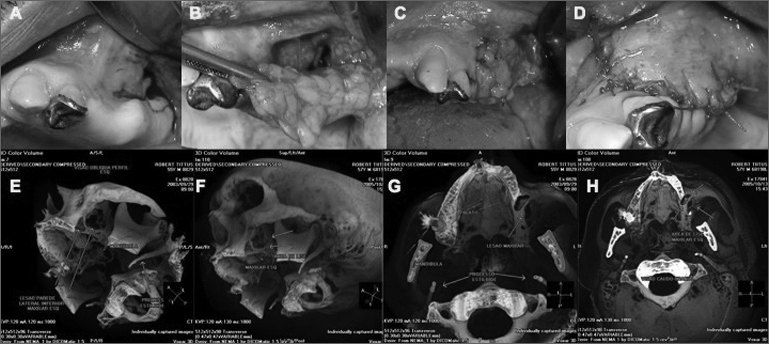
A) Incision surrounding the fistula and two horizontal relief incisions (one anterior and one posterior to the fistula). B) Exposure of the buccal adipose body, which is extended to the bone defect with no tension. C) Suturing the buccal adipose body to the rims of the palatine and vestibular mucosae. D) Suturing the mucosa over the adipose body. E) Preoperative lateral image (3D CT) of the bone defect. F) Postoperative lateral image (3D CT) of the bone defect after one year. G) Preoperative occlusal image (3D CT) of the bone defect. H) Postoperative occlusal image (3D CT) of the bone defect.

Examination of the mouth revealed a 1 cm fistula in the bottom of the upper left sulcus, which contained pus. Computed tomographic 3D reconstruction demonstrated the bone defect and its proportion (Figure 1E, 1G).

Preoperative antimicrobial therapy was started to control the infection, after which surgery was scheduled.

Anesthesia involved a block of the upper posterior and middle alveolar nerves and the greater palatine nerve. An incision was made around the fistula and two other relief incisions were also made (Figure 1A). The bone defect was visualized, the necrotic tissue was removed from the bone rims and abundant irrigation was made with saline and an ampule of rifamycin (Rifocina® 75mg/1.5ml).

An incision was made on the periosteum, the tissue was dissected and the buccal adipose body was rotated to the defect, covering it fully with no tension (Figure 1B). The buccal adipose body was sutured to the rim of the palatine and vestibular mucosa with 4/0 chrome catgut (Figure 1C). The mucosal flap was repositioned over the adipose tissue and similarly sutured. (Figure 1D).

Postoperative medication included antimicrobials for preventing infection and the usual wound care measures.

Seven days later the wound was closed, the adipose tissue was healing, and the patient reported that the symptoms were generally subsiding. Twenty days after surgery, the mucosa was well positioned over the fully healed area and there was a slight excess fat tissue, which was removed in a second procedure.

One year after surgery, computed tomographic 3D reconstruction showed that the bone defect in the lateral wall of the maxillary sinus had regressed. (Figure 1F, 1H)

## DISCUSSION

The buccal adipose body is a syssarcosis, a type of specialized fat that fills in the masticatory space, improves and dampens muscle mobility and adds to facial morphology.^4^

The advantages of using it are: a quick and simple procedure, minimum failures rates, local anesthesia, no visible scars, low morbidity and no loss of sulcus depth.^4^, ^5^

Its disadvantages are: single use, the possibility of postoperative trismus, limited use for small and mid-sized defects and no rigid support.^4^, ^5^

Epithelization takes two to three weeks; the fat acts as a basis for the growth of epithelium. Granulation tissue develops first, followed by stratified epithelium that migrates from the gingival margin. In the case report, we noted that the size of the bone defect decreased one year after buccosinusal closure, probably due to wear of the bone defect rim that may have activated repair mechanisms that were unable to close the defect completely due to its possibly critical size.

## FINAL COMMENTS

The buccal adipose body is a stable, relatively simple procedure for closing buccoinusal fistulae; the success rate is high and the postoperative period is comfortable for patients.
